# A quiescent cell population replenishes mesenchymal stem cells to drive accelerated growth in mouse incisors

**DOI:** 10.1038/s41467-017-02785-6

**Published:** 2018-01-25

**Authors:** Zhengwen An, Maja Sabalic, Ryan F. Bloomquist, Teresa E. Fowler, Todd Streelman, Paul T Sharpe

**Affiliations:** 1 Centre for Craniofacial and Regenerative Biology, Dental Institute, Kings College London, London, SE1 9RT UK; 2 Petit Institute for Bioengineering and Bioscience, Georgia Institute of Technology, Atlanta, USA

## Abstract

The extent to which heterogeneity within mesenchymal stem cell (MSC) populations is related to function is not understood. Using the archetypal MSC in vitro surface marker, CD90/Thy1, here we show that 30% of the MSCs in the continuously growing mouse incisor express CD90/Thy1 and these cells give rise to 30% of the differentiated cell progeny during postnatal development. In adulthood, when growth rate homeostasis is established, the CD90/Thy1^+^ MSCs decrease dramatically in number. When adult incisors are cut, the growth rate increases to rapidly re-establish tooth length and homeostasis. This accelerated growth rate correlates with the re-appearance of CD90/Thy^+^ MSCs and re-establishment of their contribution to cell differentiation. A population of Celsr1^+^ quiescent cells becomes mitotic following clipping and replenishes the CD90/Thy1 population. A sub-population of MSCs thus exists in the mouse incisor, distinguished by expression of CD90/Thy1 that plays a specific role only during periods of increased growth rate.

## Abstract (web summary)

Mouse incisors constantly renew from a slow cycling population of mesenchymal stem cells. Here, the authors show that upon cutting of adult incisors, a sub-population of dental mesenchymal stem cells reactivates, allowing an increased growth rate and rapid regeneration.

## Introduction

The extent to which mesenchymal stem cells (MSCs) in any single tissue or organ are a heterogeneous population remains highly contentious. Propagation of MSCs in vitro and flow cytometry based on expression of different surface proteins has suggested that different sub-populations of MSCs can be present in a single tissue^1–5^. Similarly, cell surface protein heterogeneity of perivascular cells (pericytes) that can provide a source of MSCs in many tissues has been interpreted as evidence for MSC heterogeneity^1,3–9^. In vivo, the use of genetic lineage tracing is beginning to provide evidence for different origins of MSCs^10^ and also of lineage hierarchies similar to those already known for the hematopoietic system^10,11^. Significantly however although sub-populations of MSCs may be identified from their molecular characteristics, ascribing specific functions to any such sub-populations has not been possible.

Mammalian teeth harbour MSC populations in their inner soft tissue the dental pulp^12–14^. In non-growing teeth such as human and mouse molars these cells are quiescent and only activated following extensive tooth damage^15^. In the mouse incisor however, a clearly identifiable population of continuous active MSCs can be visualized at the apical end of the tooth. These cells are required to provide a source of cells to maintain continuous growth of the incisor that is necessary to replace tissue lost from the tips during occlusion^16,17^. The continuously growing mouse incisor thus provides a highly accessible model to study stem cell behavior during growth where the cells and their niche have an obvious physical location with anatomical landmarks. Genetic lineage tracing has established that the MSC population is slow cycling, expresses Gli1 in response to Shh released from a neurovascular bundle present at the apical end of the tooth between the epithelial cervical loop^16^. This population of MSCs gives rise to rapidly dividing transit amplifying cells more distally that differentiate into two main cells types, pulp cells and odontoblasts, the specialized cells that are responsible for dentine formation. The MSCs give rise to differentiated cells throughout the adult life of the tooth at a constant rate that exactly compensates for tissue loss from the occluding tips.

In this study we show that a sub-population of MSCs is present in the incisor, characterized by expression of CD90/Thy1, whose function is to provide a source of cells only during periods of rapid growth. This population is replenished by mobilization of a stem cell reservoir population expressing Celsr1. The stimulus for this mobilization does not involve loss of mechanical forces and remains to be identified. Identification of these functional sub-populations provides new insights into the architecture of the MSC microenvironment that has implications for clinical applications that are directed towards the activation of resident stem cells.

## Results

### CD90 is expressed in a subpopulation of mesenchymal stem cells

**Fig. 1:**
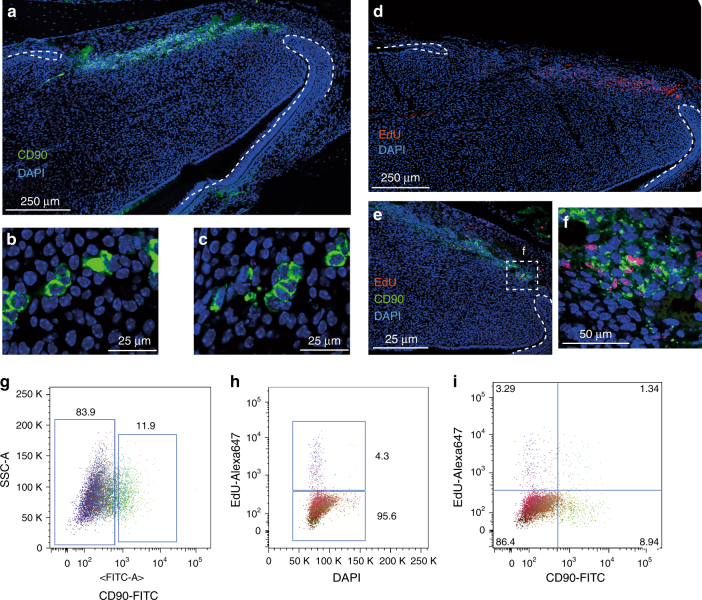
CD90/Thy1 expression in small clusters of cells in the dental mesenchyme. **a** Immuno-fluorescent staining shows CD90/Thy1 expression in the dental mesenchyme between the labial and lingual aspects of the cervical loop at the apical end of the mouse incisor on the sagittal section images. **b**, **c** High magnification images show CD90/Thy1 is expressed in small clusters of cells. **d** Long-chase EdU retaining cells occupy a similar location between the two aspects of epithelial cervical loop. Mice were given EdU for four weeks and chased for another four weeks prior to tissue collection. **e**, **f** Co-staining of CD90/Thy1 and EdU demonstrates partially co-localization. **g** FACS analysis of CD90/Thy1 expression in the postnatal (PN5) mouse dental pulp tissue showing about 12% of pulp cells express CD90/Thy1. **h**, **i** Long-chase EdU retention assay shows 4.3% of slow cycling cells are present in the incisor pulp (**e**), and about 30% of these slow cycling cells express CD90/Thy1

**Fig. 2:**
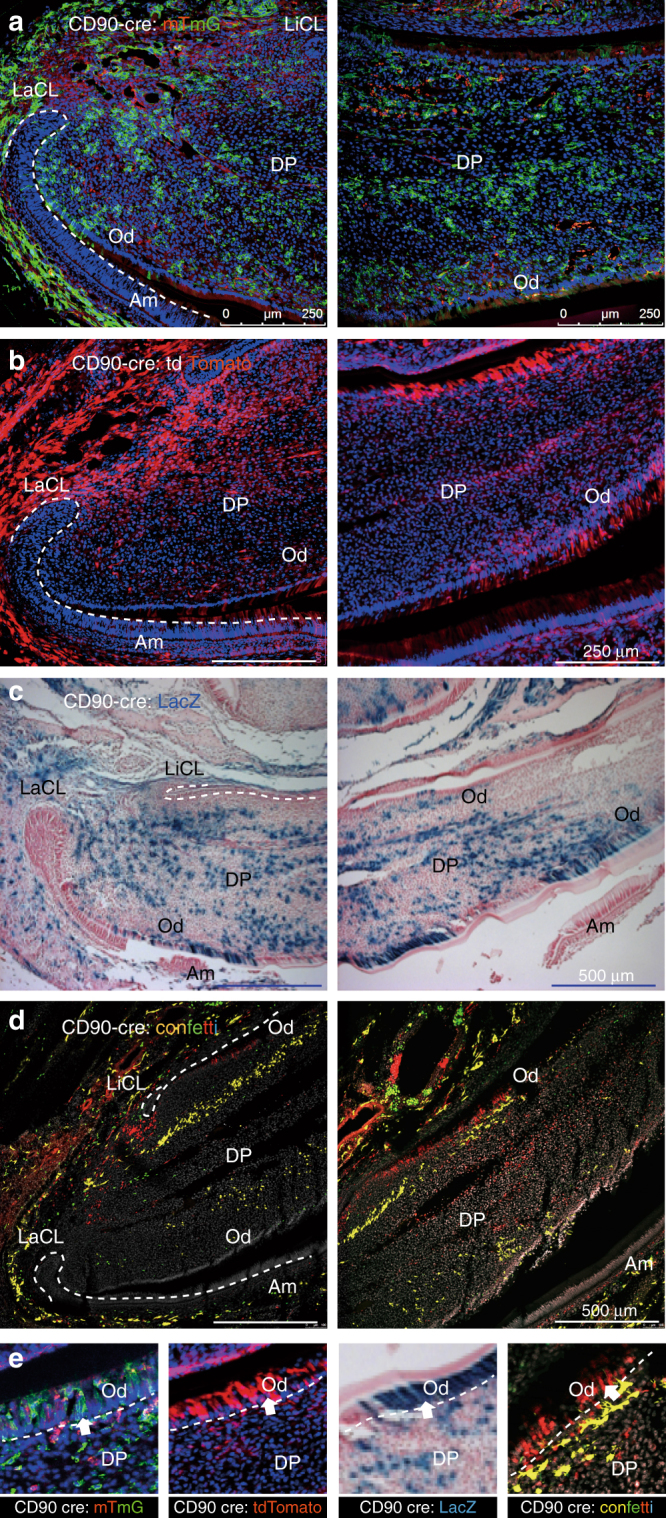
Lineage tracing of CD90/Thy1 in the postnatal mouse incisor. CD90/Thy1 cre mouse line crossed with different reporter lines, **a** Rosa26R;-mTmG, **b** Rosa26R-tdTomato, **c** Rosa26R;-LacZ, and **d** Rosa26R;-Confetti, shows CD90/Thy1 expressing cells and their progeny contribute to pulp cells and odontoblasts. **d** Confetti predominately labels single cell clones; thus each single color represents the progeny from one stem cell. **e** Enlarged region from each reporter line showing CD90/Thy1-derived odontoblasts. PN5-10 postnatal incisor samples were used in **a**–**c**, PN21 mouse incisors were used in **d**. *N* > = 5 pups each group

**Fig. 3:**
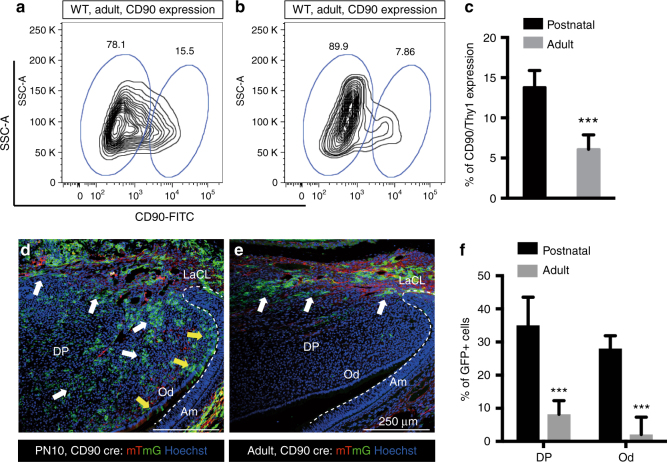
CD90/Thy1 reduced expression and contribution to the pulp cells and odontoblasts in adult incisors. **a**, **b** Flow cytometry data showing CD90/Thy1 expression is greatly reduced in the adult incisor compared to postnatal mice. **c** Quantification of CD90/Thy1 expressing cells in the mouse incisor. Data shows a 2 fold decrease in expression in adult incisors compared to postnatal. **d**, **e** Lineage tracing of CD90/Thy1 with mTmG reporter identifies the dramatic reduction of contribution to the pulp cells and odontoblasts in the adult compared to the advanced postnatal stages. White arrows show pulp cell contribution and yellow arrows show the odontoblast contribution. **f** Quantification of CD90/Thy1 contribution to the pulp cells and odontoblasts showing significantly decreased expression in adults compared with young postnatal mice. *N* > = 3 mice per group. ***P < 0.001 by Student’s *t*-test. Data presented as mean ± S.E.M

The incisor mesenchymal stem cells (MSCs) have been reported not to express many of the markers that are generally ascribed to MSCs in vitro but do express CD90/Thy1^2,17^. In the course of studying CD90/Thy1 expression in the incisor we observed a band of expressing cells co-localizing with slow cycling cells (Fig. 1a, d–f). CD90/Thy1^+^ cells were present as small clusters (Fig. 1b, c) and flow cytometry identified around 30% of the slow cycling MSCs expressed CD90/Thy1 at postnatal stages (PN5-10) (Fig. 1g–i). We next utilized a Thy1-cre mouse line with four different reporters to lineage trace the CD90/Thy1expressing cells to provide evidence that they were stem cells and could form differentiated cells types of the incisor during growth (Fig. 2). CD90/Thy1-derived cells were seen randomly scattered throughout the pulp and as odontoblasts (Fig. 2). Cell number counts of CD90/Thy1-derived pulp cells and odontoblasts suggest a sub-population of MSCs that express CD90/Thy1 and contribute to about 30% of the cell differentiation during postnatal growth/development (Fig. 3).

### CD90+ MSCs correlate specifically with accelerated growth

Following tooth eruption, the incisors in each jaw grow until they occlude with each other and the wearing/sharpening process begins that results in tissue/cell loss from the tips. At this point the growth rate is stabilized to exactly match the tissue loss and homeostasis is established. We thus looked for CD90/Thy1 expression in adult (8–10 weeks) incisors and found that expression in cells in the MSC zone was substantially reduced compared with postnatal stages (Fig. 3a–c). Interestingly, the reduction in CD90/Thy1 expressing cells was not reflected in any reduction in the number of MSCs (slow cycling Edu+ cells) which remained constant even after 1 year (Supplementary Fig. 1a–c). CD90/Thy1-cre lineage tracing confirmed the dramatic reduction of expressing stem cells in adults since very few labeled cells were detected in the pulp or odontoblast layer (Fig. 3d–f). Thus in adult incisors the population of CD90/Thy1^+^ MSCs that contributes to incisor growth during the eruption process is depleted.

**Fig. 4:**
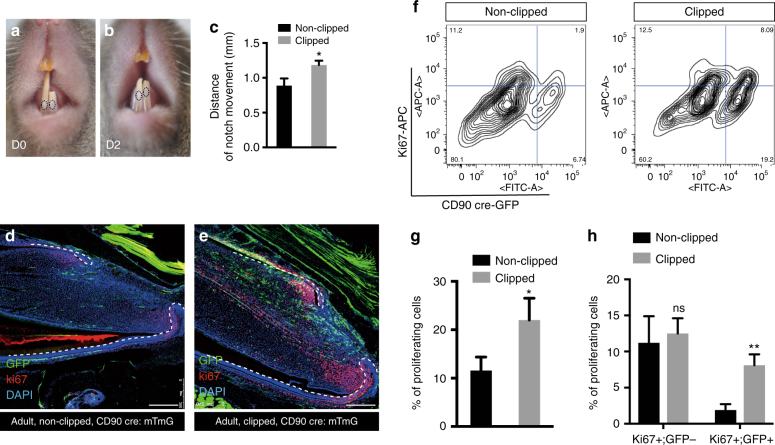
Rapid expansion of CD90/Thy1 expressing MSCs contributes to re-establishment and homeostasis after adult incisor clipping. **a**–**c** 2 days after clipping (D2), notch movement on the unclipped incisor and clipped incisor was measured using a digital caliper, indicating faster growth of clipped incisor compared to the unclipped control (*n* = 5, 8–10 weeks old adult mice). **d**, **e** Immuno-fluorescent staining of the cell proliferation marker, Ki67 on CD90/Thy1 cre; mTmG mice with clipped incisors showing higher proliferation and increased contribution to the pulp cells and odontoblasts from CD90/Thy1-derived cells than in non-clipped incisors. **f**–**h** FACS analysis and quantification shows significantly increased numbers of proliferating cells in clipped incisors with almost all the increase in proliferating cells being accounted for by CD90/Thy1^+^ cells (Ki67 + ;GFP+), while the Ki67+;GFP− cell population remains constant. Bar is 250 µm. *P < 0.05 and **P < 0.01 by Student’s *t*-test. Data presented as mean ± S.E.M

**Fig. 5:**
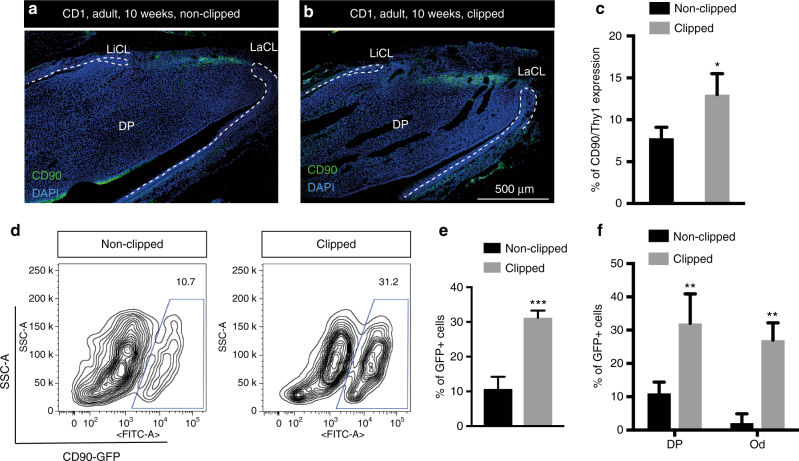
Increased CD90/Thy1 expression is localized to the incisor MSC region after clipping. **a**, **b** Immunofluorescent staining of CD90/Thy1 identifies expression localized to the region in dental pulp between the epithelial cervical loop and no expression is detected in the TAC region 2 days after clipping. **c** Quantification of CD90/Thy1 expression in clipped incisors compared to unclipped incisors (*N* > = 3 per group, 10 week adult mice). **d**, **e** FACS analysis of CD90/Thy1 cre; mTmG mice shows a three fold increase in GFP + cells from clipped incisors compared to unclipped. **f** Quantification of GFP + cells in the pulp cells and odontoblasts on sagittal sections. N > = 5 mice per group. *P < 0.05, **P < 0.01 and ***P < 0.001 by Student’s *t*-test. Data presented as mean ± S.E.M

To understand why this sub-population of MSCs is not required during adult homeostatic growth, but does contribute to growth during eruption, we used a simple clipping approach to stimulate the growth rate. We removed the uppermost 1/3–1/2 of the erupted part of incisors by clipping and made a notch in the enamel close to the junction with the gingival soft tissue to mark the tooth. The movement of the notches was then measured and compared between clipped and unclipped incisors during the following 2 days. As a result of the clipping we observed the growth rate of the incisors to be increased significantly (Fig. 4a–c). We next stained tissue sections for proliferation markers to determine if the population of fast cycling transit amplified cells was increased in response to clipping that might account for the increased growth. We noted a drastic increase in the number of Ki67-marked cells in the clipped incisors 2 days after clipping compared with unclipped controls (Fig. 4d, e). The increase in cell proliferation in the transit amplifying population could have been caused by an intrinsic increase in proliferation rate or extrinsically by the provision of more cells from the MSC population. We thus used flow cytometry to analyze the proportion of Ki67^+^ cells derived from CD90^+^ cells before and after clipping (Fig. 4f). The number of proliferating cells almost doubled after 48 h following clipping (Fig. 4g) and the increase in Ki67 + cells was fully accounted for by CD90/Thy1^+^ cells (Fig. 4h). Significantly the number of Ki67^+^;CD90^−^ cells remained constant before and after clipping, indicating that the increase in proliferating cell numbers was attributable to the provision of new CD90^+^ cells (Fig. 4h). To eliminate the possibility that clipping induces CD90/Thy1 expression in proliferating cells we carried out CD90/Thy1 immunolocalisation and found that the increase in + cells was resticted to the MSC region and no + cells were observed outside this region (Fig. 5a–c). Clipping of the incisor thus stimulates a reappearance of CD90/Thy1 expressing cells in the MSC population that in turn can account for the increased number of transit amplifying cells. To determine if the “new” population of CD90/Thy1expressing MSCs contributed to cell differentiation we carried out clipping of Thy1-cre; mTmG incisors. CD90/Thy1^+^ cells were present in the pulp and the odontoblast layer and contributed to about up to 30% of the odontoblasts during the accelerated growth period following clipping (Fig. 5d–f). The re-appearance of CD90/Thy1 expressing cells following clipping suggested either a massive upregulation of proliferation of the few remaining detectable cells or re-population of the CD90/Thy1 expressing stem cell pool from another cell source.

### A reservoir cell population forms CD90 cells

**Fig. 6:**
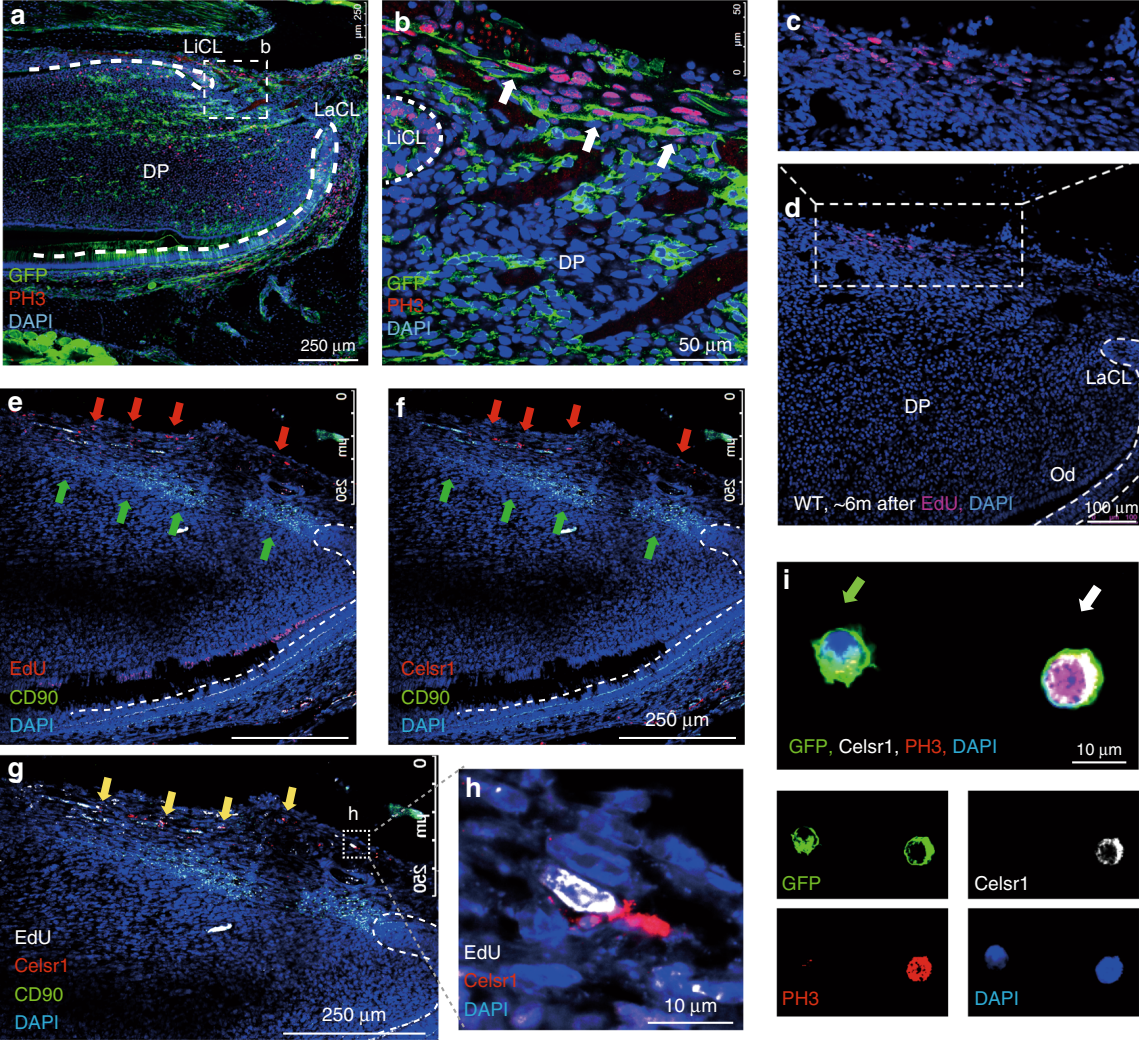
Quiescent cells act as a reservoir and mobilize to form stem cells. **a**, **b** PH3 immuno-staining of sections of CD90/Thy1 cre; mTmG mice shows proliferating cells become detectable in the mesenchyme between the two aspects of the cervical loop after incisor clipping. **c**, **d** Detection of EdU incorporating cells that had been labeled during embryonic development from E2.5 to E17.5 and chased for over 6 months, showing a small population of EdU+ cells distributed in a line at then most proximal end of incisor mesenchyme. **e** Co-staining with CD90/Thy1 and EdU labeled quiescent cells shows CD90/Thy1 cells are located in the mesenchyme adjacent to the EdU labeled cells, red arrows are EdU + and green arrows are CD90/Thy1^+^ cells. **f** Co-staining with CD90/Thy1 and Celsr1 shows CD90/Thy1 cells occupy the same location adjacent to the Celsr1 expressing cells. **g** Triple staining with CD90/Thy1, Celsr1 and EdU shows CD90/Thy1 expressing cells are located adjacent to the EdU and Celsr1^+^ cells, yellow arrows and **h** enlarged region showing the co-localization of EdU+ ; Celsr1^+^ cells. **i** Immuno-staining of FACS sorted GFP + cells from clipped CD90/Thy1-cre;mTmG mouse incisors with Celsr1 and PH3 on cytospin slides, identifies GFP+ ;Celsr1^+^;PH3^+^ triple positive (white arrow,) and GFP^+^ ;Celsr1^−^;PH3^−^ cells (green arrow). Bar 250 µm in (**a**), (**e**), (**f**), and (**g**). 50 µm in b, 100 µm in d and 10 µm in (**h**) and (**i**)

Normally, proliferation rates of the MSCs are very low (slow cycling cells) and mitoses cannot be detected in these cells. PH3 antibody staining from mitotic cells in the MSC zone of clipped incisors revealed a distinct zone of mitotic cells immediately proximal to the CD90/Thy1 expressing cells (Fig. 6a, b). These mitotic cells were located at the extreme most proximal end of the incisor mesenchyme in a line parallel to the CD90/Thy1^+^ MSC zone (Fig. 6a, b). Since in all previous nucleoside incorporation regimes to detect slow cycling MSCs and rapid cycling transit amplifying cells we had never observed dividing cells at the location we decided to determine if the cells at the location were quiescent prior to clipping. In order to identify the origin of these proliferating cells we injected EdU for two weeks into pregnant mothers carrying E2.5–E17.5 embryos reasoning that these cells must divide at some point in embryogenesis. We then chased the nucleoside for 6 months in the offspring before sacrifice to identify any cells that had retained the label and thus remained quiescent into adulthood. A small population (0.6%) of nucleoside retaining cells (Fig. 6c, d; Supplementary Fig. 1d), distributed in a line, were visible at the most proximal extent of the incisor mesenchyme indicative of a population of quiescent cells proximal to the slow cycling CD90/Thy1^+^ MSCs (Fig. 6e). In the haematopoietic system a population of quiescent cells marked by expression of Celsr1 (Flamingo) has been identified that provides a reservoir of cells that are mobilized to form stem cells following depletion of the haematopoietic stem cells^18^. We thus analyzed the location of Celsr1 expressing cells by immunohistochemistry in the growing incisor and observed a small population distributed in a line in the most proximal mesenchyme, clearly distinct from CD90/Thy1-expressing cells and observed co-localization of Celsr1 with Edu-labeled quiescent cells (Fig. 6f–h). In order to provide evidence that Celsr1^+^ quiescent cells are stimulated by clipping to divide and become CD90/Thy1^+^ MSCs we searched for double positive cells using cytospins. We were able to observe CD90/Thy^+^;Celsr1^+^;PH3^+^ cells alongside CD90/Thy1^+^;Celsr1^−^;PH3^−^cells that could also be visualized in vivo (Fig. 6i; Supplementary Fig. 2). Such triple positive cells were never observed in the absence of clipping. Celsr^+^ cells, quiescent cells and cells that undergo mitosis following clipping all occupy the same location immediately proximal to the MSCs. Although lineage tracing of these cells was not possible and thus their conversion to MSCs cannot as yet be confirmed, this spatial localization and acquisition of CD90/Thy1 expression and cell division following clipping, together with the similarities with the haematopoietic system, suggest these cells act a reservoir of cells held in reserve for times when the stem cell population needs to be increased.

### Evolutionary conservation of Celsr1+ slow cycling cells

While the rodent incisor serves as a model for continuous renewal of dental cells, many vertebrates such as squamates and fishes have the capacity to replace their entire teeth throughout life^19,20^. We assayed activity of Celsr1 in conjunction with pulse-chase experiments to identify label-retaining cells in Lake Malawi cichlid fishes, a well-studied animal model of dental development and replacement. Animals were bathed in BrdU for 1 week starting at 4dpf, the point at which the primary and first generation of successional teeth are formed. BrdU was then removed and animals were chased for 100 days, allowing for several generations of tooth replacement. Animals were then sacrificed and double-label immunostaining experiments were carried out for Celsr1 and BrdU. In the mesenchymal dental papilla, co-labeled Celsr1^+^;BrdU^+^ cells were observed in the tip of the papilla and surrounding the cervical loops, as well as in the follicle (Supplementary Fig. 1e, f). The co-localization of Celsr1 expressing cells with label-retaining cells observed in cichlid fish tooth replacement and mouse incisor continuous growth, is suggestive of a likely evolutionary conserved mechanism for maintenance of a reservoir of mesenchymal stem cell precursors.

### Loss of occlusion does not accelerate growth

**Fig. 7:**
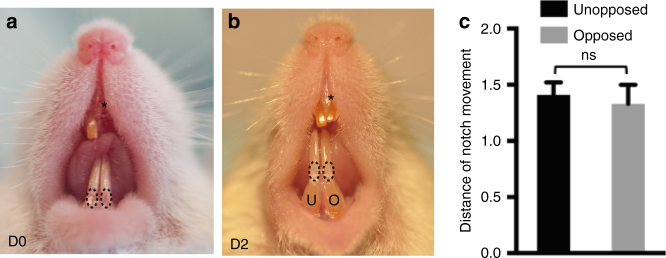
Measurement of growth rates of opposed incisors after clipping. **a**, **b** Mice incisors were clipped on the upper right side and a marker notch made on both lower incisors just above the gingiva. The notch movements were measured 2 days after clipping. Asterisk indicates clipped incisor. **c** Quantification of notch movements shows no significant difference in growth rates of unopposed (U) and opposed (O) incisors. *N* > = 5 mice per group. *NS* not significant by Student’s *t*-test. Data presented as mean ± S.E.M

The position of the clipping distal to the location of the MSC niche (including both stem cells and transit amplifying zones), suggests that the effect on the MSCs is indirect and that a signal induced by the clipping is able to stimulate the expansion of the CD90/Thy1^+^ sub-population of MSCs by mobilization of the quiescent cells. Since clipping prevents occlusion the obvious mechanism to consider for this signal transmission is one that is induced by a change in mechanical load on the incisors. In order to test this idea, we simply clipped one incisor and left the opposing incisor unclipped and notched it to measure growth rate. In this scenario the unclipped incisor has lost its contact with its opposing incisor and thus mechanical load is reduced. The growth measurements showed that there was no difference in growth of the unopposed incisor with an opposed incisor (Fig. 7). Prevention of occlusion does not therefore stimulate accelerated growth, suggesting that mechanical load is unlikely to be the source of the growth stimulus. There thus appears to be a signal (at present of unknown identity) that is transmitted down the incisor from the clip site to the quiescent/MSC cell populations to mobilize the expansion of the CD90/Thy1^+^ cells to accelerate growth.

## Discussion

CD90/Thy1 is an archetypal membrane marker of mesenchymal stem cells (MSCs) that is used along with other markers to define cells as MSCs in vitro^21,22^. Expression of CD90/Thy1 in MSCs in vivo has rarely been described and thus the extent to which expression of CD90/Thy1 is indicative of an MSC in vivo and indeed in vitro is questionable. The Shh pathway transcription factor Gli1 is expressed in all incisor MSCs, that are located in a neurovascular bundle at the proximal end of the incisor^3^. Gli1+ MSCs are in part derived from neuronal glia^2^. We show here that CD90/Thy1 is expressed in a subset of incisor MSCs that contribute to 30% of the odontoblasts and pulp cells during early postnatal development. Thus although in vitro, most MSCs are described as expressing CD90/Thy1, in the incisor, most MSCs do not express CD90/Thy1 and thus this begs the question of whether CD90/Thy1 expression in vitro occurs as a result of clonal expansion and selection of more rapidly dividing cells. CD90/Thy1 expressing cells are barely detectable once the incisor has erupted when growth rate homeostasis is established. When the tips of the incisors are clipped, the CD90/Thy1 expressing cells reappear and once again contribute to the formation of odontoblasts and pulp cells. This reappearance corresponds to a period of accelerated growth, stimulated by the clipping, that is necessary to rapidly restore incisor length so that they can occlude. The newly formed CD90/Thy1^+^ MSC population is solely responsible for providing cells for the accelerated growth and CD90/Thy^−^ cells do not increase their contribution. The MSC sub-population of MSCs marked by CD90/Thy1 expression thus plays a specialized role in contributing cells for rapid growth phases of the incisor and during homeostasis plays little if any role. The reappearance of CD90/Thy1 expressing cells following clipping occurs via proliferation of a quiescent cell population that express Celsr1. This very small population of cells resides proximal to the MSCs and appears to generate CD90/Thy1 expressing cells. Of particular interest is the expression of Celsr1 in a population of cells in the bone marrow that act as a reservoir to provide new hematopoietic stem cells following their depletion^18^. Our observation of the expression of Celsr1 in label retaining cells in the mesenchyme of replacement teeth of cichlids hints at a possible generic role for these cells as reservoirs for stem cell production in adult tissues.

The mobilization of Celsr1^+^ cells in the incisor following clipping does not appear to be stimulated by the loss of the mechanical forces of occlusion. Clipping clearly results in the transmission of some as yet unidentified signal to the reservoir cells that selectively stimulates their proliferation

This is the first identification of discrete sub-populations of MSCs, marked by differential gene expression (markers) that have a specific role in increasing growth rates in a tooth can provide the basis for the understanding similar populations in other tissues and organs. It also has implications for in vivo mobilization of stem cells to enhance tissue repair where targeting of specific “reserve” cell populations to accelerate growth and repair may be more effective than generic activation of all stem cells in a niche.

## Methods

### Mouse strains and lineage tracing

All animal work was carried out according to Home Office guidelines in the UK under project license number PPL70/7866 and approved by the KCL animal ethics committee. Wild type CD1 mice were from CRL (Charles River Laboratory, UK).

CD90/Thy1-cre, R26R-LacZ, R26-tdTomato, R26-mTmG and R26R-Confetti reporter line (Brainbow 2.1) were from JAX (Stock Number 006143, 003309, 007905, 007576 and 013731 respectively).

### Immunofluorescence

Immunofluorescence staining used standard protocol on 12 µm sagittal cryosections of mouse incisors. Anti-mouse CD90.2-FITC (eBioscience 11-0903, 1:50 for staining on sections and 1:400 for cells), Anti-rabbit Celsr1 antibody (Millipore ABT119, 1:200), Anti-rabbit Ki67 antibody (Abcam ab15580, 1:100), Anti-rabbit Phospho Histone 3 (EMD Millipore, 06-570, 1:100), Anti-mouse Phospho Histone 3 (Abcam, ab14955, 1:300), Anti-chicken GFP (Abcam, ab13970, 1:1000). Secondary antibodies used 1:250 for immuostaining on sections and 1:400 for FACS analysis: goat anti-rabbit IgG (H + L) Alexa Fluor 635 (Life technologies, A31576), goat anti-chicken IgG (H + L) Alexa Fluor 488 (Life technologies, A11039), goat anti-mouse IgG (H + L) Alexa Fluor 647 (Life technologies, A21235), rabbit anti-mouse IgG (H + L) Alexa Fluor 546 (Life technologies, A11060), goat anti-mouse IgG (H + L) Alexa Fluor 633 (Life technologies, A21052), donkey anti-rabbit IgG (H + L) Alexa Fluor 594 (Life technologies, A21207), Goat anti-rabbit IgG (H + L) Alexa Fluor 488 (Life technologies, A11008). Hoechst 33342 (Invitrogen 62249, 1:500) was used for DNA staining. Slides were then mounted using Citifluor™ AF1 (Citifluor Ltd., AF1-100) and cover-slipped for microscopy. CD90/Thy1 cre: Confetti incisors were sectioned to 20–30 µm and nuclei were stained with TO-PRO 3-Iodide (Thermo Fisher Scientific, T3605).

### EdU incorporation and staining

For slow cycling cell labeling, EdU (3.3 µg/body weight) was injected into PN5 pups for 4 weeks continuously and traced for 2 months to one year. For quiescent cell labeling, EdU (3.3 µg/body weight) was injected into pregnant mice every day during embryonic stage from E2.5 to E17.5. Litters of pups were then left for 6~7 months before scarifie and jaws were collected, fixed and decalcified prior to cryo-embedding and sectioning. EdU was detected by Click-iT EdU Alexa Fluor 647 Imaging kit (Invitrogen C10340) according to the protocol.

### Flow cytometry

Mouse incisor pulp tissue was freshly dissected and dissociated into single cell suspension by TrypLE Express Enzyme (Thermo Fisher Scientific 12604013). Cells were then fixed and stained by Click-iT EdU Alexa Fluor 647 Flow Cytometry Assay kit (Invitrogen C10424) according to the protocol before being subjected to Flow cytometry. FACS analysis was carried on BD Fortessa cell analyser and data was analyzed by BD FACSDiva 6.1.3 or flowjo software.

### Incisor clipping experiments

The mice were anaesthetized with a combination of fentanyl-fluanisone (Hypnorm, VetaPharm Ltd.) and midazolam (Hypnovel, Roche) in sterile water in the 1:1:2 ratio, 10 ml/kg i.p. Clipping of incisors was performed on adult mice by removing a third of the erupted part of the lower incisor. Both incisors were notched above the gumline using a diamond cylinder bur (Henry Schein, 9791465) in a high speed handpiece to monitor growth rate changes in clipped vs. control incisors. Measures were taken by a digital caliper (Absolute digimatic caliper, Mitutoyo, 500-196-20).

### Cell counting and statistics

Statistical analysis was done using GraphPad Prism (GraphPad Software Inc.) and Microsoft Office 2016 program package (Microsoft Corporation). Paired Student’s *t*-test was used to calculate statistical significance. Values of *p* < 0.05 were considered statistically significant. Minimum of 3–5 animals were used for each experiment. Control for incisor growth measurement was a contralateral unclipped but notched lower incisor of the same animal. Unclipped animals’ teeth were used as immunohistological controls to exclude any effects of notching and increased occlusal forces on the adjacent contralateral lower tooth following clipping of the other incisor.

### Microscopy and imaging

Confocal microscopy used Leica TCS SP5 system and imaging processing analysis used LAS AF imaging software. Confetti reporters’ tissues were imaged, as previously described^23^. CD90-driven EGFP was excited using the argon laser 488 nm line (fluorescence collected between ∼498 and 510 nm); for EYFP 514 nm line (fluorescence collected between ∼530 and 560 nm; for RFP a DPSS laser emitting at 561 nm (fluorescence collected between ∼590 and 650 nm) and ECFP was excited using laserline at 458 nm (fluorescence collected between ∼420 and 491 nm).

### Cichlids

*Metriaclima zebra* [MZ] Malawi cichlids were housed in re-circulating aquarium systems at 28 °C (GIT) for embryo production. Specimens 4dpf were bathed in 2% BrdU in-vivo labeling reagent (Invitrogen 00-0103) in 200 mL of fish water at 28 °C. 1 mL aliquots of BrdU solution were added every 24 h for 6 additional days during the “pulse” period. Embryos were rinsed twice and moved to fresh water at 28 °C in a re-circulating aquarium system (GIT). Embryos were sacrificed after 100 days chase and fixed in 10% NBF at RT at 4 °C, rinsed in PBS, and decalcified for a period of 48–72 hrs in a mild acid (0.1 M EDTA) at RT.

Specimen were processed through a graded series of EtOH (25, 50, 75, 100, 100%) and then washed in xylene for 3 h before being incubated 60 °C/embedded in paraffin for sectioning on a Thermo Scientific Microm HM355S microtome at 5 µm. Slides were dried for 24 h at 42 °C and rehydrated through xylene and a graded series of EtOH for incubation in blocking solution (BS-3% goat serum, 1% bovine serum, 0.1% Triton ×100) for 1 hr at RT. Slides were then incubated O/N in a 1:100 dilution of anti-rabbit primary antibody (rabbit ant-celsr1 (Anaspec- 55716)) and the provided blocking solution containing nuclease enzyme at 4 °C. Slides were rinsed 2 × 1 h in PBS and incubated in secondary antibodies at 1:400 HRP conjugated goat anti-rabbit IgG (Molecular Probes) and Alexa Fluor 568 goat anti-mouse IgG2a (Molecular Probes) in BS at RT. Unbound secondary antibody was washed 2 × 1 h in PBS and the HRP signal was amplified using a 488-tyramide chemistry signal amplification kit (Molecular Probes). Slides were rinsed 2 × 1 h, mounted with 50:50 glycerin:Vectashield, and imaged using a Zeiss 710 confocal microscope.

### Data availability

The authors declare that all data supporting the findings of this study are available within the article and its supplementary information files or from the corresponding author upon reasonable request.

## Electronic supplementary material

Supplementary Information
