# Supplementary material for: A quiescent cell population replenishes mesenchymal stem cells to drive accelerated growth in mouse incisors
Source: Nat Commun. 2018 Jan 25;9:378. doi: 10.1038/s41467-017-02785-6 (PMC5785476; doi:10.1038/s41467-017-02785-6)
Supplement: Supplementary file 1 — Supplementary Information [file 41467_2017_2785_MOESM1_ESM.pdf]

Supplementary figure 1. Long-chase EdU/Brdu labelling cells in mice and cichild fishes.

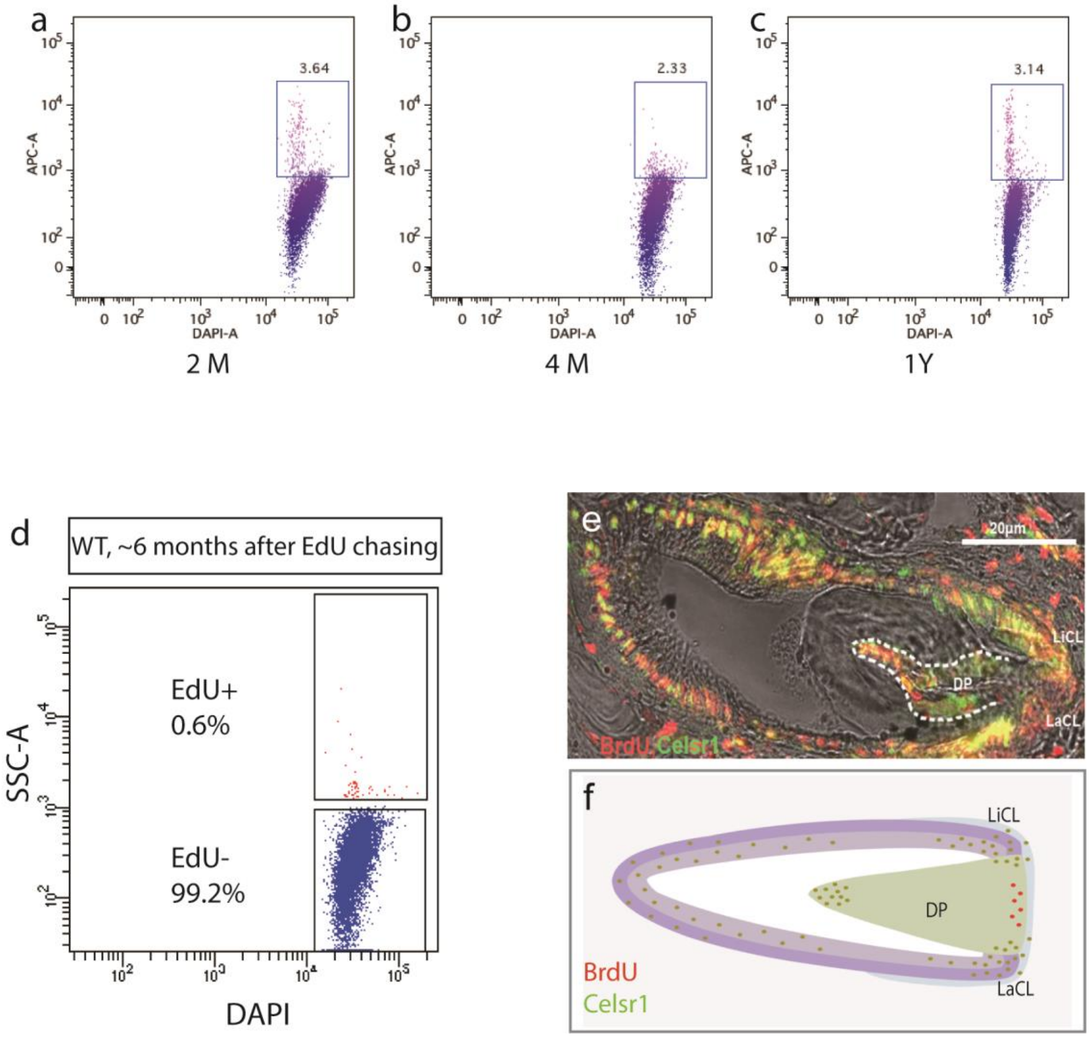

a-c EdU was continuously given to postnatal P5 pups for four weeks and chased for up to one year. Tissues were collected at 2 months, 4 months and one year after chasing prior to flow cytometry analysis. FACS results show the percentage of EdU labelled cells remains constant from 2 months to one year after chasing. **d** Labelling of quiescent cells in the incisor pulp via EdU administration to pregnant mice from E2.5 to E17.5 and chased for six months before tissue collection. FACS analysis shows only 0.6% of EdU labelled quiescent cell are detected in the whole population of incisor pulp cells. **e** Cichlid fishes were bathed in Brdu for 1 week starting at 4dpf and chased for 100 days before tissue collection. Co-localisation of Celsr1 and

label-retaining (BrdU+) cells at the tip of the papilla and surrounding the cervical loop in addition to the follicle of cichlid teeth and, **f,** illustration of Celsr1 expression and BrdU localisation in a cichlid replacement tooth.

Supplementary figure 2. ***In vivo*** **identification of GFP+;Celsr1+;PH3+ triple labelling cells in CD90/Thy1 cre;mTmG mice after clipping.**

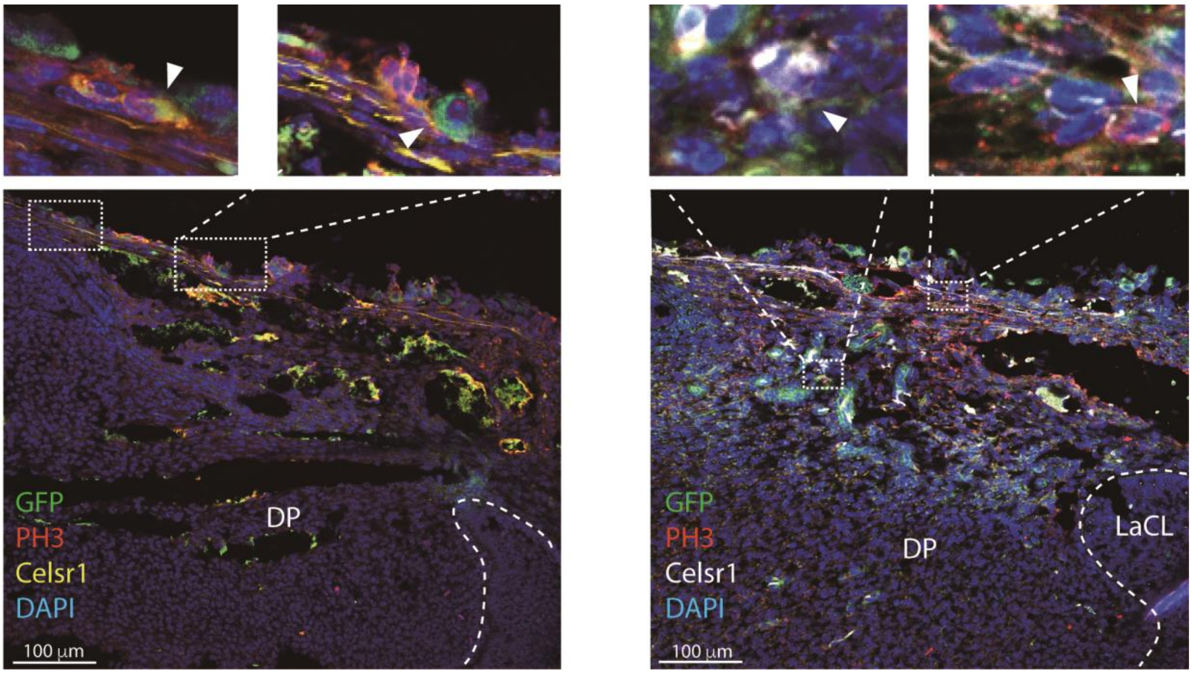

Identification of quiescent cell mobilisation into stem cells to accelerate growth after clipping, Examples of immunofluorescent staining of Celsr1 and PH3 on sagittal sections of CD90/Thy1 cre;mTmG mice 2 days after clipping. CD90/Thy1-GFP+;Celsr1+;PH3+ triple labelling cells were detected in the proximal end of incisor mesenchyme showing in the enlarged field of images and indicate with white arrows (CD90/Thy1 GFP is green, PH3 is red, Celsr1 is yellow on the left panel image and white on the right hand image, DAPI is blue for nuclear staining).
